# K_ATP_ channels in cerebral hemodynamics: a systematic review of preclinical and clinical studies

**DOI:** 10.3389/fneur.2024.1417421

**Published:** 2024-07-03

**Authors:** Hassan Ali Suleiman Daoud, Lili Kokoti, Mohammad Al-Mahdi Al-Karagholi

**Affiliations:** ^1^Department of Neurology, Danish Headache Center, Copenhagen University Hospital- Rigshospitalet, Copenhagen, Denmark; ^2^Department of Clinical Medicine, Faculty of Health and Medical Sciences, University of Copenhagen, Copenhagen, Denmark; ^3^Department of Neurology, Nordsjaellands Hospital- Hilleroed, Hilleroed, Denmark

**Keywords:** CBF, cerebral arteries, ATP-sensitive potassium channels, migraine, stroke

## Abstract

Cumulative evidence suggests that ATP-sensitive potassium (K_ATP_) channels act as a key regulator of cerebral blood flow (CBF). This implication seems to be complicated, since K_ATP_ channels are expressed in several vascular-related structures such as smooth muscle cells, endothelial cells and pericytes. In this systematic review, we searched PubMed and EMBASE for preclinical and clinical studies addressing the involvement of K_ATP_ channels in CBF regulation. A total of 216 studies were screened by title and abstract. Of these, 45 preclinical and 6 clinical studies were included. Preclinical data showed that K_ATP_ channel openers (KCOs) caused dilation of several cerebral arteries including pial arteries, the middle cerebral artery and basilar artery, and K_ATP_ channel inhibitor (KCI) glibenclamide, reversed the dilation. Glibenclamide affected neither the baseline CBF nor the baseline vascular tone. Endothelium removal from cerebral arterioles resulted in an impaired response to KCO/KCI. Clinical studies showed that KCOs dilated cerebral arteries and increased CBF, however, glibenclamide failed to attenuate these vascular changes. Endothelial K_ATP_ channels played a major role in CBF regulation. More studies investigating the role of K_ATP_ channels in CBF-related structures are needed to further elucidate their actual role in cerebral hemodynamics in humans.

**Systematic review registration:** Prospero: CRD42023339278 (preclinical data) and CRD42022339152 (clinical data).

## Introduction

Cerebral hemodynamics including cerebral blood flow (CBF) and cerebral vascular tone are vital parameters contributing to brain homeostasis (1). Dysregulation of cerebrovascular hemodynamics is involved in the pathogenesis of several neurological disorders such as stroke and migraine (2, 3). The molecular mechanisms involved in the modulation of cerebral hemodynamics are complex and not entirely comprehended.

**Figure 1:**
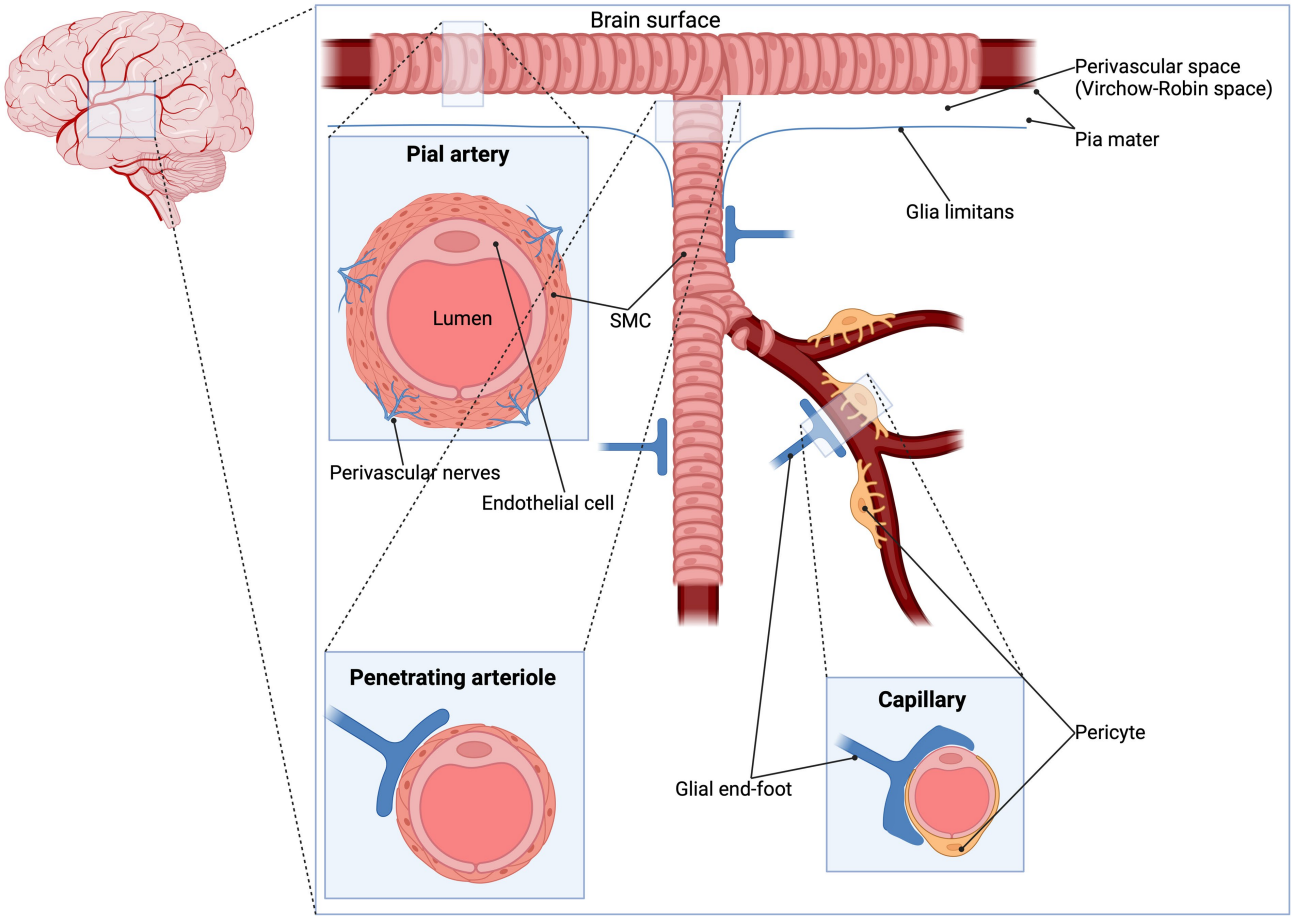
Pial artery, penetrating arteriole and capillary. The pial arterial vasculature (also known as pial collaterals or leptomeningeal anastomoses) consists of smaller arteries and arterioles that connects the three major supplying the arteries of the cerebrum: the anterior cerebral artery, the middle cerebral artery and the posterior cerebral artery (13). The pial arteries are intracranial arteries on the surface of the brain within the pia-arachnoid (leptomeninges) or glia limitans (the outmost layer of the cortex composed of glial end-feet), surrounded by cerebrospinal fluid (14) and give rise to smaller penetrating arterioles (15). An important difference in vessel architecture which might influence the CBF regulation is the number of SMC layers: penetrating arterioles contain one layer of smooth muscle while smaller pial arteries contains two to three layers of smooth muscle (16). Since K_ATP_ channels are expressed in SMC, it is expected that these channels have a higher impact in pial arteries. To date, no studies did compare the effect of KCO/KCI between these types of vessels. CBF, cerebral blood flow; K_ATP_, ATP-sensitive potassium; KCI; K_ATP_ channel inhibitor KCO; K_ATP_ channel opener; SMC, smooth muscle cell.

**Figure 2:**
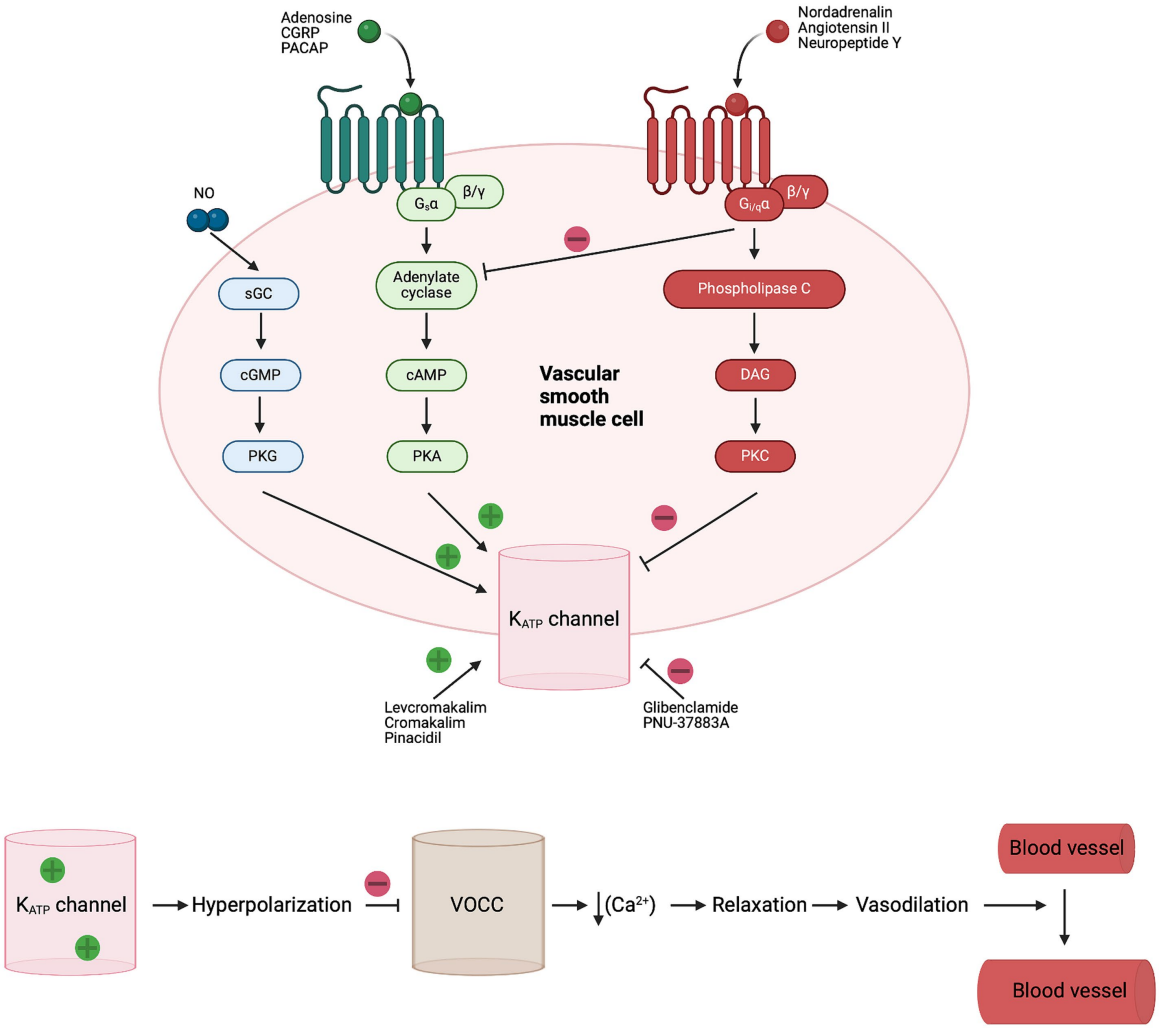
Signaling pathway and opening of K_ATP_ channels in vascular SMC. Numerous endogenous vasodilators activate K_ATP_ channels in SMC through adenylate cyclase and PKA phosphorylation. While endogenous vasoconstrictors inhibit K_ATP_ channels in SMC through DAG and PKC phosphorylation. Activation of K_ATP_ leads to hyperpolarization and closing of voltage-operated Ca^2+^ channels (VOCC), followed by relaxation of SMC and increased blood flow (17). CGRP, calcitonin gene-related peptide; DAG, diacylglycerol; Gs, G-protein-coupled receptor alpha s; Gi/q, G-protein-coupled receptor alpha i/q; sGC, soluble guanylate cyclase; K_ATP_, ATP-sensitive potassium; NO, nitric oxide; PACAP, pituitary adenylate cyclase activating polypeptide; PKA, protein kinase A; PKC, protein kinase C; PKG, protein kinase G; SMC, smooth muscle cell; VOCC, voltage-operated Ca^2+^ channels.

Evidence from preclinical and clinical studies implicates ATP-sensitive potassium (K_ATP_) channels in the regulation of CBF and the cerebral vascular tone (4–6). K_ATP_ channels are vastly expressed at several structures of the vasculature such as arteries, penetrating arterioles and the complex mesh of capillaries. Specifically, K_ATP_ channels are present in smooth muscle cells (SMCs), endothelial cells (ECs) and pericytes (7–12) (Figure 1). K_ATP_ channels link the cellular metabolic state to the plasmalemma’s electrophysiology. They are activated during ischemia and hypoxia, causing potassium efflux, hyperpolarization and subsequently vasodilation (17–19) (Figure 2).

The intricate mechanisms underpinning the involvement of K_ATP_ channels in the regulation of cerebral hemodynamics have not been systematically reviewed. Here, we systemically review preclinical and clinical studies addressing the expression of K_ATP_ channel in the cerebral vasculature, and their involvement in CBF regulation and cerebral vasodilation.

## Methods

We searched PubMed and EMBASE for articles assessing the role of K_ATP_ channel in the cerebral vasculature. The search was conducted on 29 January 2024, and the search string was (“K_ATP_ channels” [MeSH Terms] OR “K_ATP_ channel” [All Fields] OR “ATP sensitive potassium channel” [All Fields] OR “K_ATP_ channel expression” [All Fields] OR “K_ATP_ channel knockout” [All Fields] OR “ATP sensitive potassium channel expression” [All Fields] OR “ATP sensitive potassium channel knockout” [All Fields] AND “cerebral blood flow” [MeSH Terms] OR “cerebral blood flow” [All Fields] OR “brain blood flow” [All Fields] OR “blood flow, brain” [All Fields] OR “cerebral circulation” [All Fields] OR “cerebral circulations” [All Fields] OR “flow, brain blood” [All Fields] OR “circulation, cerebrovascular” [All Fields] OR “cerebrovascular circulation” [All Fields]).

### Selection criteria and study inclusion

An *a priori* systematic review protocol was developed. The full protocol can be obtained from the corresponding author upon reasonable request. Two study protocols were registered in Prospero [ID-numbers: CRD42023339278 (preclinical data) and CRD42022339152 (clinical data)]. We followed the Preferred Reporting Items for Systematic Reviews and Meta-analyses (PRISMA) reporting guidelines and the recommendations from the Cochrane Collaboration (20). The population, intervention, comparison, outcome, and study design (PICOS) approach was chosen as follows: study design, sample characteristics of the sample, intervention, comparator and outcomes.

After removing duplicates, two investigators (HASD and LK) independently screened articles, first by title and abstract and then full text to confirm eligibility for this review. The references of the included studies were also screened. Any disagreements between the investigators were resolved through discussion. If the conflict remained, a third investigator (MMK) made the final decision. Studies were restricted to English language and both preclinical and clinical studies investigating K_ATP_ channel opener (KCO) or K_ATP_ channel inhibitor (KCI; Table 1) and their effects on CBF and the diameter of cerebal arteries were included. Reviews, meta-analysis, conference proceedings and case reports were excluded. For each included study, the following data were extracted: article information (title, authors, and journal), study design, characteristics of the sample intervention, technique, substances used, and outcomes. No formal meta-analysis was planned.

**Table 1:** An overview of KCOs and KCIs included in the studies.

**KCOs**	
	Levcromakalim
	Cromakalim
	Diazoxide
	Pinacidil
	Aprikalim
	Iptakalim
	Nicorandil
	Y-26763
**KCIs**	
	Glibenclamide
	BaCl_2_
	Tolbutamide
	Glyburide
	Hydroxylysine
	PNU-37883
	PNU-37883A

KCI; K_ATP_ channel inhibitor KCO; K_ATP_ channel opener.

## Results

**Figure 3:**
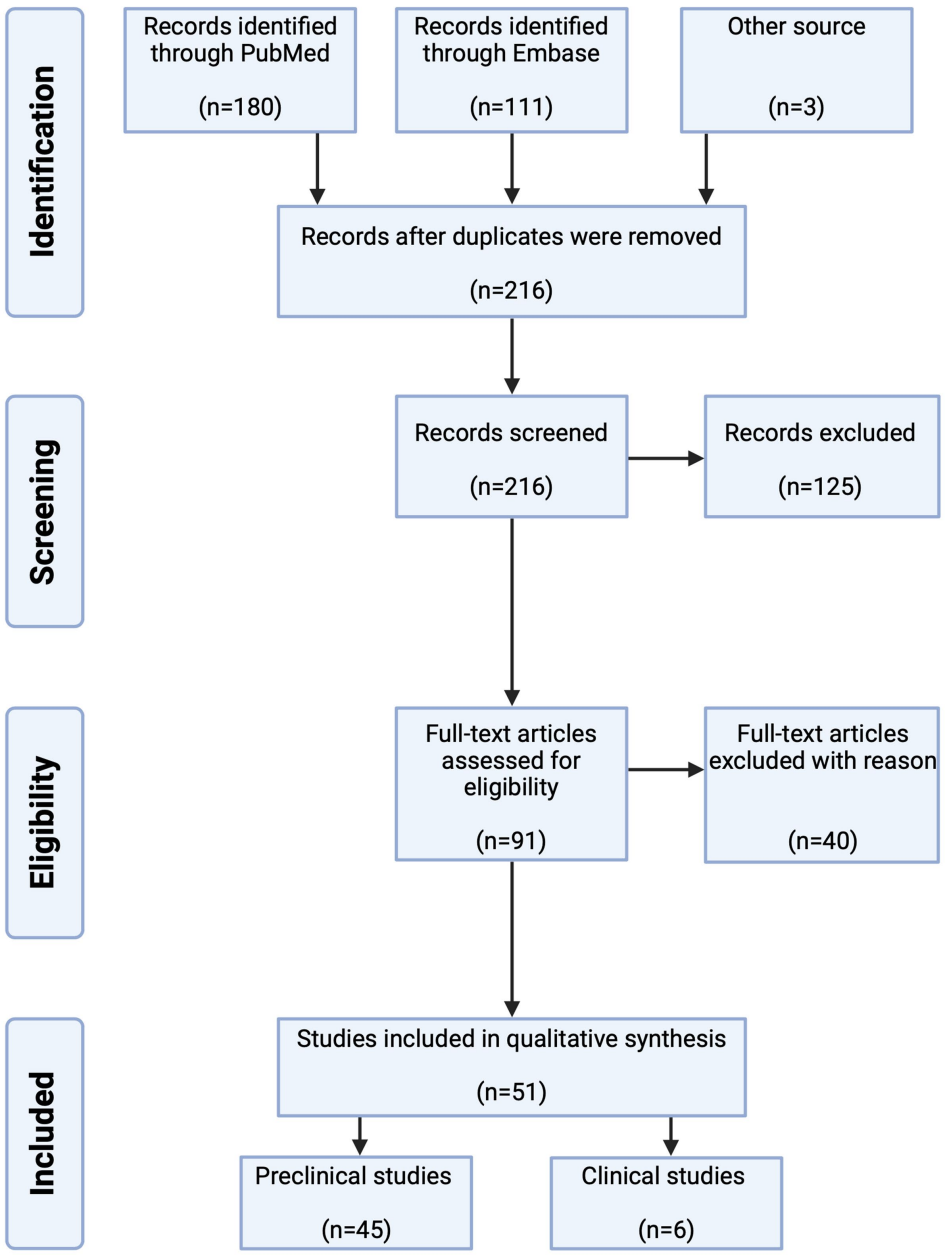
Flowchart of search strategy.

The database search identified 294 citations of which 78 were duplicates. A total of 216 studies were screened by title and abstract and 91 were full text screened. Of these, 51 studies were included, 45 preclinical (35 studies *in vivo*, seven studies *ex vivo*, two studies *in vivo* and *ex vivo* and one study *in vivo* and *in vitro*) and six clinical studies (Figure 3). Preclinical and clinical data are summarized in Tables 2, 3, respectively.

**Table 2:** Summary of preclinical studies.

N	Author	Purpose of the study	Substance(s) and dose(s)	Study population (n)	Method	Main outcome(s)	Conclusion
1	Armstead W et al. (5)	To investigate the effect of ischemia/hypoxia on K_ATP_ induced pial artery dilation.	Cromakalim (10^−8^–10^−6^ M).	Pigs (*n* = 55)	Diameters of pial arteries were measured using a video microscopy through a cranial window over the parietal cortex.	Ischemia or hypoxia blunted dilation of pial arteries induced by cromakalim.	Ischemia or hypoxia impaired K_ATP_ channel mediated cerebrovasodilation.
2	Ca et al. (21)	To investigate the vascular activity of mastoparan in the cerebral circulation and the role of K_ATP_ channel activation.	Mastoparan (10^−8^–10^−6^ M).	Pigs (*n* = 24)	Diameters of pial arteries were measured with video microscaler through a cranial window over the parietal cortex.	Mastoparan, a pertussis toxin-sensitive G protein, induced pial artery dilation which was blunted by co-administered glibenclamide.	G-protein activation elicited cerebrovasodilation through interaction with K_ATP_ channels.
			Glibenclamide (10^−6^ M).				
3	Ann et al. (22)	To investigate the effect on fluid percussion brain injury (FPI) on K_ATP_ channel activity.	Cromakalim (10^−8^–10^−6^ M).	Pigs (*n* = 144)	Diameters of pial arteries were measured using a video microscaler through a cranial window over the parietal cortex.	Cromakalim induced dilation of pial arteries which was blunted for at least 72 h post FPI in the newborn pigs and at least 4 h post FPI in the juvenile pigs, respectively.	Newborn pigs were more sensitive to traumatic vascular injury than the juvenile pigs.
					FPI was produced using a pendulum to strike a piston on a saline-filled cylinder.	K_ATP_ channel function was impaired to a greater extent and for a longer time period in the newborn vs. the juvenile pig.	
4	Armstead et al. (23)	To investigate the role of heat shock protein (HSP) in the modulation of K^+^ channel induced pial artery dilation after FPI.	Cromakalim (10^−8^ M).	Pigs (*n* = 30)	Diameters of pial arteries were measured with a video microscaler through a cranial window over the parietal skull.	Cromakalim and CGRP induced dilation of pial arteries.	HSP-27 and HSP-70 contributed to modulation of K^+^ channel induced pial artery dilation.
			CGRP (10^−6^ M).			Under non-FPI, co-administration of exogenous HSP-27 blunted dilation to cromakalim and CGRP. However, co-administration of exogenous HSP-70 potentiated dilation to cromakalim and CGRP.	
			HSP-27 (1 μg/mL).		FPI was produced using a pendulum to strike a piston on a saline-filled cylinder.		
			HSP-70 (1 μg/mL).			FPI increased the concentration of HSP-27 in cerebrospinal fluid and decreased the concentration of HSP-70.	
5	Armstead et al. (24)	To investigate whether K^+^ channel functional impairment arising after FPI is prevented by phenylephrine in a sex-dependent manner.	Cromakalim (10^−8^–10^−6^ M).	Pigs (*n* = 70)	Diameters of pial arteries were measured with a video microscaler through a cranial window over the parietal cortex.	Cromakalim dilated pial arteries, that was impaired after FPI, more in males than in females.	Phenylephrine prevented impairment of K_ATP_ channel-mediated cerebrovasodilation after FPI in females.
			Phenylephrine (1 μg/kg/min).		FPI was produced using a pendulum to strike a piston on a saline-filled cylinder.	After cromakalim, phenylephrine prevented reductions in cerebrovasodilation in females, but reduced the dilation in males.	
6	Armstead et al. (25)	To investigate whether vasopressin generates superoxide anion (O_2_^−^) in a cyclooxygenase dependent manner which could link vasopressin release to impaired K_ATP_ channel-induced pial artery dilation after FPI.	Cromakalim (10^−8^–10^−6^ M).	Pigs (*n* = 90)	Diameters of pial arteries were measured using a video microscaler through a cranial window over the parietal cortex.	Under non-brain injury, vasopressin co-administered with cromakalim, diminished dilation of pial arteries induced by cromakalim.	Vasopressin blunted K_ATP_ channel mediated cerebrovasodilation after FPI.
			Vasopressin (40 pg./mL).		FPI was produced using a pendulum to strike a piston on a saline-filled cylinder.	Cromakalim induced pial artery dilation was attenuated following FPI.	
7	Pastor et al. (26)	To investigate whether inhaled nitric oxide (NO) prevents impairment of cerebrovasodilation in response to cromakalim after FPI.	Cromakalim (10^−8^–10^−6^ M).	Pigs (*n* = 60)	Diameters of pial small arteries were measured using ANOVA for repeated measures through a cranial window over the parietal skull.	FPI impaired pial small artery dilation in response to cromakalim.	Inhaled NO prevented impairment of cerebral autoregulation after traumatic brain injury through protection of K^+^ channel function.
					FPI was produced using a pendulum to strike a piston on a saline-filled cylinder.	Inhaled NO prevented loss of pial artery dilation in response to cromakalim.	
8	Wei et al. (27)	To investigate whether blockade of K_ATP_ channels in pial arterioles inhibits vasoconstriction from hypocapnic alkalosis.	Glyburide (1 μM).	Cats (*n* = 15)	Diameters of pial arterioles were measured using an image-splitting device attached to a microscope through a cranial window over the parietal cortex.	Hypocapnic alkalosis induced vasoconstriction of pial arterioles that was blocked by glyburide, hydroxylysine or L-NNA.	Inhibition of K_ATP_ channel in pial arterioles inhibited the vasoconstriction from hypocapnic alkalosis.
			Hydroxylysine (1 μM).				
			N^G^-nitro-L-arginine (L-NNA) (250 μM).			All the drugs did not cause significant changes in baseline diameter.	
9	Nnorom et al. (28)	To investigate whether K_ATP_ channels play a role in neonatal cerebral dilation in response to hypercapnia.	Pinacidil (10^−5^ M).	Pigs (*n* = NR)	Diameters of pial arterioles were measured using a video microscope through a cranial window over the parietal cortex.	Pinacidil or hypercapnia caused dilation of pial arterioles.	Hypercapnia activated K_ATP_ channels leading to cerebral dilation of arterioles.
						Glibenclamide blocked the dilation to pinacidil and hypercapnia.	
			Glibenclamide (10^−7^–10^−6^ M).		Hypercapnia was induced by ventilation with 5% or 10% CO_2_ and 21% O_2_.	However, glibenclamide alone had no effect on baseline diameters.	
10	Bari et al. (29)	To investigate the effects of ischemia on cerebral responses to arterial hypoxia and adenosine.	Adenosine (10^−5^–10^−4^ M).	Pigs (*n* = 22)	Diameters of pial arterioles were measured using intravital microscopy through a cranial window over the parietal cortex.	Ischemia did not alter dilation of cerebral arterioles to arterial hypoxia and to adenosine.	Cerebral dilation to hypoxia and adenosine was maintained after ischemia.
						Dilation of cerebral arterioles to arterial hypercapnia was reduced by ischemia.	
			Glibenclamide (10^−6^–10^−5^ M).		Ischemia was achieved by increasing intracranial pressure.	Glibenclamide reduced dilations of cerebral arterioles to adenosine but did not change baseline diameters.	
11	Patel et al. (30)	To investigate the effects of endothelin-1 (ET-1) on cystathionine δ -lyase catalyzed brain H_2_S production.	ET-1 (10^−12^–10^−8^ M).	Pigs (*n* = 50)	Diameters of pial arterioles were measured with video microscaler through a cranial window over the parietal cortex.	ET-1 caused dilation of pial arterioles, an effect which was completely blocked by glibenclamide.	H_2_S mediated the vasodilator effect of ET-1 in the cerebral circulation via a mechanism that involved activation of K_ATP_ channels in vascular SMC.
			Glibenclamide (10^−7^ M).			ET-1 increased H_2_S production by the brain via cystathionine δ -lyase activation.	
12	Bari et al. (31)	To investigate whether cerebral vasodilation induced by aprikalim is dependent on production of NO.	Aprikalim (10^−8^–10^−6^ M).	Piglets (*n* = 40)	Diameters of pial arterioles were measured using a video microscaler through a cranial window over the parietal cortex.	Aprikalim induced dilation of pial arterioles. However, L-NAME attenuated this dilation.	Aprikalim-induced dilation of pial arterioles is mediated partly by NO.
			Glibenclamide (10^−5^ M).				
			N^G^-nitro-L-arginine methyl ester (L-NAME) (15 mg/kg).			Glibenclamide did not alter baseline diameter.	
13	Lida et al. (32)	To investigate the effects of isoflurane and sevoflurane on pial arterioles via K_ATP_ channel activation.	Isoflurane	Dogs (*n* = 24)	Diameters of pial arterioles were measured using a video micrometer through a cranial window over the parietal cortex.	Inhalation or topical application of either isoflurane or sevoflurane induced dilation of pial arterioles and glibenclamide attenuated the dilation.	Dilation of pial arterioles appeared to be activated by K_ATP_ channels.
			Sevoflurane				
			Glibenclamide (10^−7^–10^−5^ M).		Systemic (inhalation) and topical administration of isoflurane and sevoflurane.		
14	Wei et al. (4)	To investigate the role of K^+^ channels in the vasodilator action on pial arterioles.	Pinacidil (10^−7^–10^−6^ M).	Cats (*n* = 54)	Diameters of pial arterioles were measured with a Vickers image splitting device through a cranial window over the parietal cortex.	Pinacidil and cromakalim dilated pial arterioles which was inhibited by glyburide.	K_ATP_ channels played a role in the vasodilation of pial arterioles.
			Cromakalim (10^−7^–10^−6^ M).				
			Glyburide (1 μM).				
15	Faraci et al. (33)	To investigate whether aging is associated with impaired dilation of cerebral arterioles in response to activation of K_ATP_ channels.	Aprikalim (1–10 μM).	Rats (*n* = 7)	Diameters of cerebral arterioles were measured using a video microscope through a cranial window over the parietal cortex.	Aprikalim dilated cerebral arterioles that was similar in adult and old rats.	Activation of K_ATP_ channels were preserved during aging.
16	Parfenova et al. (34)	To investigate the effects of sulforaphane in intact cerebral circulation.	Glibenclamide (10^−7^ –10^−6^ M).	Pigs (*n* = 28)	Diameters of pial arterioles were measured using intravital microscopy through a cranial window.	Glibenclamide blocked the cerebral vasodilator responses to sulforaphane.	Sulforaphane-induced cerebral vasodilation was dependent on K_ATP_ channel.
			Sulforaphane (10^−6^ M–10^−3^ M or 0.4 mg/kg).			Glibenclamide did not change the baseline diameters of pial arterioles.	
17	Mayhan et al. (35)	To investigate the effects of K_ATP_ channel activation on diameter of pial arterioles and whether diabetes mellitus alters responses of pial arterioles to activation of K_ATP_ channels.	Aprikalim (0.1–10 μM).	Rats (*n* = 29)	Diameters of pial arterioles were measured using a video image-shearing device through a cranial window over the parietal cortex.	Aprikalim produced dose-related dilation of pial arterioles in non-diabetic rats but produced constriction or/and minimal dilation of pial arterioles in diabetic rats.	K_ATP_ channels regulated cerebral arterioles and were impaired during diabetes mellitus.
						The dilation of pial arterioles in non-diabetic rats was abolished by glibenclamide.	
			Glibenclamide (1 μM).			Glibenclamide did not change the baseline diameters of pial arterioles.	
18	Horinaka et al. (36)	To investigate K_ATP_ channel blocker on the CBF response to insulin-induced hypoglycemia.	Glibenclamide (1–2 μM).	Rats (*n* = NR)	Infusion of glibenclamide in cisterna magna.	Glibenclamide had no significant effect on CBF in normoglycemic rats.	K_ATP_ channel was an important component of the mechanisms of the CBF response to hypoglycemia.
					CBF was determined by autoradiographic [^14^C] iodoantipyrine (IAP) method (37).	Glibenclamide blocked the increases in CBF in hypoglycemia in a dose-dependent manner.	
19	Takanori et al. (38)	To investigate whether K_ATP_ channels participate in tonic regulation of CBF.	Glibenclamide (1–10 μM).	Rats (*n* = NR)	Infusion of glibenclamide in cisterna magna.	Glibenclamide tended to lower baseline CBF in the cerebellar lobules, cerebellar cortex, pontine nuclei and spinal trigeminal nucleus.	K_ATP_ channel could play role in the tonic regulation of baseline CBF.
					CBF was determined by autoradiographic [^14^C] IAP method (37).		
20	Tomiyama et al. (39)	To investigate CBF during hemodilution and hypoxia.	Glibenclamide (19.8 g).	Rats (*n* = 48)	Infusion of glibenclamide in cisterna magna.	Hypoxia induced a greater increase in CBF in the forebrain, cerebellum and brain stem than hemodilution.	K_ATP_ channels did not contribute to increasing CBF during hemodilution.
					CBF of the forebrain, cerebellum and brain stem were calculated by the indicator fractionation method with variables syringe flow, tissue weight etc.	Glibenclamide treatment attenuated the increase of CBF during hypoxia but not hemodilution.	Intravascular P_O2_ was an important regulator of cerebral vascular tone.
21	Takaba et al. (40)	To investigate the effect of K_ATP_ channel on CBF in hypertensive rats.	Y-26763 (25 mg/L).	Rats (*n* = 26)	Co-administration of Y-26763 (intracarotid infusion) and glibenclamide (intravenous infusion).	Infusion of Y-26763 increased CBF, which was inhibited by glibenclamide.	K_ATP_ channel could contribute to the regulation of CBF.
						However, glibenclamide did not alter the baseline CBF.	
			Glibenclamide (20 mg/kg).		CBF were measured using laser-Doppler flowmetry through a cranial window over the parietal cortex.	The response to Y-26763 was significantly impaired in hypertensive rats.	K_ATP_ channel was diminished in hypertensive rats.
22	Golonov et al. (41)	To investigate tolbutamide and glibenclamide effects on regional cerebral blood flow (rCBF) and arterial pressure elicited by hypoxemia.	Glibenclamide (5–200 pmol).	Rats (*n* = 15)	Microinjection of tolbutamide and glibenclamide into rostral ventrolateral medulla (RVL).	Tolbutamide and glibenclamide into RVL increased rCBF and facilitate elevations of rCBF induced by hypoxia.	K_ATP_ channels could mediate hypoxic excitation of oxygen-sensing RVL neurons.
			Tolbutamide (300 pmol in 20 nL).		rCBF were measured with a laser-Doppler flowmeter through a cranial window over the parietal cortex.		
23	Erdos et al. (42)	To investigate the dynamics of the rCBF changes during noxious stimulation in the thalamus and in the sensory cortex.	Glibenclamide (10 μg/rat).	Rats (*n* = 10)	Laser-Doppler flowmetry measured cortical and thalamic blood flow through a cranial window over the sensory cortex and in the medial part of the thalamus.	Noxious stimulation increased both cortical and thalamic blood flow, which was attenuated by glibenclamide.	CBF was adjusted during noxious stimulation, and this regulation involved activation of K_ATP_ channels.
					Noxious stimulation with an instrument (S44 stimulator), where sciatic nerve was electrically stimulated.		
24	Andreas et al. (43)	To investigate endothelium-derived factors on capillary ECs and K_ATP_ channels effects on capillary flow regulation and neurovascular coupling.	Pinacidil (5 mM).	Mice (*n* = NR)	*In vivo* 4D two-photon microscopy measured the regulation of microvascular flow in somatosensory cortex.	Pinacidil induced dilation of penetrating arterioles, capillaries and precapillary sphincters.	K_ATP_ channels was found in pericytes and precapillary sphincters and had a key role for blood flow control.
						PNU-37883 abolished this vasodilator effect.	
			PNU-37883 (0.5–2.5 mM).		Pericytes responses to contractile and vasodilatory signals were measured with imaged diameter changes of penetrating arterioles, capillaries and precapillary sphincters.	Capillary blood flow was regulated primarily by pericytes and precapillary sphincters.	
25	Toyoda et al. (44)	To investigate whether K_ATP_ channels regulate CBF autoregulation during hypotension.	Levcromakalim (10^−6^ M).	Rats (*n* = 20)	Diameters of basilar artery and large and small branches from the basilar artery were measured using a video-analyzer through a cranial window over the ventral brain stem.	During hypotension, levcromakalim induced dilation of the diameters of all three vessels.	K_ATP_ channels played an essential role in the regulation of CBF to the brain stem during hypotension, mediated by compensatory dilation of small arteries, but not larger arteries.
			Glibenclamide (10^−6^−10^−5^ M).		CBF to the ventral brain stem were measured by laser-Doppler flowmetry.	Glibenclamide impaired the dilator response of small arterioles but did not impaired the dilation of large arterioles or the basilar artery.	
26	Toyoda et al. (45)	To investigate regional differences and age-related changes in the contribution of K_ATP_ channels to vasodilator responses in the brain stem circulation.	Levcromakalim (10^−7^–10^−5.5^ M).	Rats (*n* = 28)	Diameters of the basilar artery and its branches were measured through a cranial window over the ventral brain stem using a microscope equipped with a TV-camera coupled to a video monitor.	Levcromakalim and Y-26763 increased the diameter of the basilar artery and its branches which was abolished by application of glibenclamide in both adult and aged rats.	No regional heterogeneity in vasodilator response in adult rats to K_ATP_ channel openers whereas dilator response of the large arteries due to activation of K_ATP_ channels is impaired in aged rats.
			Y-26763 (10^–7.5^−10^−6^ M).			The dilator responses of the branches, but not the basilar artery, were smaller in aged rats.	
			Glibenclamide (10^−6^ M).				
27	Liu et al. (46)	To investigate rCBF response to K_ATP_ channel in Alzheimer’s disease (AD) from three age groups.	Diazoxide (5 mg/kg).	Mice (3xTgAD, wild type and with Presenilin-1 mutation) (*n* = 48–78).	rCBF were measured using laser-Doppler flowmetry through a cranial window over the region supplied by the left MCA.	Diazoxide increased rCBF in young, middle-aged and old wild type mice as well as young 3xTgAD mice.	The age-exacerbated impairment of the rCBF response to diazoxide was associated to progression of A β pathology in AD brains.
						Diazoxide response to rCBF was reduced in middle-aged and old 3xTgAD mice.	
			Glibenclamide (20 μM).				
						The effect of diazoxide was abolished by glibenclamide.	
28	Liu et al. (47)	To investigate whether diazoxide modulates CBF in AD.	Diazoxide (5 mg/day).	Mice (3xTgAD) (*n* = NR)	CBF were measured using laser-Doppler flowmetry on the surface of thinner skull over the region supplied by MCA.	Diazoxide increased rCBF in 3xTgAD mice.	Diazoxide can be a therapeutic potential drug in the treatment of AD.
29	Kotoda et al. (48)	To investigate the effect of nicorandil on CBF.	Nicorandil (1, 5, or 10 mg/kg).	Mice (*n* = 48)	CBF were measured using laser Doppler flowmeter through a cranial window over the region supplied by left MCA.	1 mg/kg nicorandil increased CBF while blood pressure and heartrate remained unaltered.	K_ATP_ channel was involved in CBF regulation.
			Glibenclamide (5 mg/kg).			This effect was inhibited by co-administration of either glibenclamide or L-NAME.	
			L-NAME (3 mg/kg).			However, nicorandil at higher doses (5 and 10 mg/kg) decreased CBF by decreasing blood pressure.	
30	Takaba et al. (49)	To investigate the effect of K_ATP_ opener on focal cerebral ischemia.	Y-26763 (24 μg/kg).	Rats (*n* = 24)	Trombotic occlusion of the distal MCA was produced photochemically.	The infarct volume was smaller in Y-26763-treated group than in the control group.	Activation of K_ATP_ channel appeared to be neuroprotective in focal cerebral ischemia.
					rCBF were measured by laser-Doppler flowmetry through a cranial window.	Y-26763 did not affect CBF before and after the occlusion.	
					After 3 days, the brain was dissected into slices and infarct volume of each rat was calculated as the product of the infarct times the 2-mm thickness of each section.	However, the beneficial effect of Y-26763 may be due to a direct action on neuron instead of its vasodilation effect.	
31	Nguyen et al. (50)	To investigate the mechanisms responsible for K^+^ dilation of resistance-size cerebral arteries.	Pinacidil (10 μM).	Rats (*n* = NR)	An intact MCA was dissected from the brain and the cerebral arterioles were separated from the parenchyma.	BaCl_2_ and glibenclamide reduced dilations in cerebral arterioles and in the basilar artery induced by pinacidil.	SMCs were activated by a K_ATP_ channels.
			Glibenclamide (1 μM).		SMCs were isolated from basilar artery and patch-clamp recordings were performed.		
			BaCl_2_ (10 μM).				
32	Guo et al. (51)	To investigate the impacts of iptakalim on pericyte contraction in stroke.	Iptakalim (10 mg/kg).	Mice (*n* = NR)	MCA occlusion (MCAO) were performed.	Iptakalim significantly promoted recovery of CBF after cerebral ischemia, reperfusion and inhibited pericytes contraction.	Iptakalim could improve microvascular disturbance by inhibiting pericyte contraction after ischemic stroke.
					Laser speckle imaging to illuminate the pial microcirculation.	Furthermore, iptakalim improved cerebral microcirculation.	
					Brain tissue were sliced and placed in a collagen-gel contraction assay to demonstrate cultured pericytes.		
33	Zimmermann et al. (52)	To investigate mechanisms underlying the diminished sensitivity of cerebral arteries in diabetic mellitus rats to K_ATP_ channel openers.	Pinacidil (10^−9^–10^−5^ M).	Rats (*n* = NR)	MCA was dissected, and endothelium were removed.	Pinacidil and levcromakalim dilated MCA from both control and diabetic rats.	Diabetes mellitus resulted in a diminished response to K_ATP_ channel openers.
						However, MCA from diabetic rats were less sensitive to the drugs.	
			Levcromakalim (10^−9^–10^−5^ M).		MCA diameter was measured with a video dimension analyzer.	Dilations to K_ATP_ openers were reduced by endothelium removal.	
34	Horiuchi et al. (53)	To investigate whether K_ATP_ channels are involved in acidosis-induced dilation of cerebral arterioles.	Glibenclamide (3 μM).	Rats (*n* = 48)	Cerebral arterioles from MCA were cannulated and diameters were measured with an inverted microscope.	Acidosis-induced dilations of the cerebral arterioles which was inhibited by either BaCl_2_ or glibenclamide.	Acidosis stimulated K_ATP_ channels resulting in dilation of cerebral arterioles.
						Glibenclamide did not alter the baseline diameters of cerebral arterioles.	
			BaCl_2_ (30 μM).		The endothelium from the arterioles were removed.	The dilation was significantly attenuated after endothelial impairment.	
35	Janigro et al. (54)	To investigate the effects of K_ATP_ opening on endothelium-dependent regulation of cerebrovascular tone.	Nicorandil (1 μM).	Rats (*n* = 52)	Cerebral arterioles were separated from the parenchyma.	K_ATP_ openers nicorandil or pinacidil induced cerebrovasodilation by directly acting on vascular SMC and by causing ECs to release NO.	K_ATP_ agonist caused a pronounced vascular SMC-mediated and a lesser NO and endothelium-dependent vasodilation.
			Pinacidil (1–10 μM).		The arterioles were cannulated with extra- or intraluminal application of drugs while measuring vessel diameter changes using a video analyzer.	Extraluminal application of nicorandil or pinacidil caused a more pronounced glibenclamide-sensitive vasodilation than applied intraluminally.	
			Glibenclamide (5–100 μM).		To test if the vasodilation was mediated by endothelial NOS activation: Vessels were pretreated with NOS inhibitors L-NNA or N^G^-monomethyl-L-arginine (L-NMMA).	Glibenclamide applied either extra- or intraluminally did not affect baseline vessel diameter.	
36	Kinoshita et al. (55)	To investigate whether K_ATP_ channels play a part in vasodilator responses in cerebral microvessels.	Levcromakalim (3 · 10^−8^–3 · 10^−7^ M).	Rats (*n* = NR)	Diameters of intact cerebral arterioles were measured by a video-microscopy.	Levcromakalim induced dilation of the cerebral parenchymal arterioles which was abolished by glibenclamide or lidocaine but not by BaCl_2_.	Lidocaine could impair beneficial vasodilator responses mediated via K_ATP_ channels.
			Glibenclamide (5 · 10^−6^ M).				
			BaCl_2_ (10^−5^ M)				
			Lidocaine (10^−5^–3 · 10^−5^ M).		Arterioles were pretreated with prostaglandin $F_{2\alpha }$ .		
37	Nakahata et al. (56)	To investigate whether K_ATP_ channels contribute to cerebral vasodilation mediated by mild hypercapnia.	Levcromakalim (3 · 10^−8^–3 · 10^−7^ M).	Rats (*n* = NR)	Brain was removed, sliced and placed in a perfusion chamber.	Mild hypercapnia (CO_2_ = 50 mmHg) and levcromakalim induced significant dilation in the cerebral parenchymal arterioles, which was completely abolished by glibenclamide.	K_ATP_ channels played a crucial role in vasodilator responses produced by mild hypercapnia.
					Diameters of intact cerebral parenchymal arterioles were measured using computerized video-microscopy.		
			Glibenclamide (5 · 10^−6^ M).		Arterioles were pretreated with prostaglandin $F_{2\alpha }$ .		
38	Movahed et al. (57)	To investigate the effects of hypoxia on vasodilator responses to K_ATP_ channel opener and NO-donor, S-nitroso-N-acetylpenicillamine (SNAP).	Levcromakalim (0.01–10 μM).	Pigs (*n* = 58)	Basilar artery was dissected.	Levcromakalim or SNAP induced concentrations-dependent dilations under both standard and hypoxic condition.	SNAP was a more effective vasodilator than levcromakalim during hypoxia.
					Tension experiments.	Under hypoxic conditions, vasodilation induced by levcromakalim was not significantly affected, which is more pronounced in SNAP-induced dilations.	
			Glibenclamide (10 μM).		The artery was precontracted by ET-1.	Glibenclamide attenuated levcromakalim-induced vasodilation.	
39	Li et al. (58)	To investigate the effects of adenosine on the physiology of retinal pericytes.	Adenosine (5 μM).	Rats (*n* = NR)	Patch-clamp electrophysiology to monitor the whole-cells currents of intact pericytes located on micro-vessels, isolated from retinas.	Hyperpolarization of retinal pericytes is due to the activation of K_ATP_ channels by adenosine or pinacidil, an effect which was blocked by glibenclamide.	Regulation of K_ATP_ channels allowed adenosine to serve as a vasoactive signal in the retinal microvasculature.
			Glibenclamide (100 nM).				
			Pinacidil (5 μM).		Membrane potential measurement.		
			Barium (3 mM).				
40	Sancho et al. (11)	To investigate whether ECs and pericytes in CNS capillaries expresses K_ATP_ channels.	Glibenclamide (20 μM).	Mice (*n* = NR)	Patch-clamp electrophysiology to measure whole cell current in isolated capillary ECs, pericytes and SMCs from cerebral pial arteries.	Pinacidil, adenosine or CADO, respectively, increased CBF.	K_ATP_ channels had an important role in capillary ECs and pericytes in the regulation of CBF.
			Pinacidil (10 μM).			Pinacidil, adenosine or CADO, respectively, in capillary ECs and pericytes caused membrane potential hyperpolarization, an effect that was reversed by glibenclamide and PNU-37783.	
			Adenosine analog (CADO) (1 μM).			Glibenclamide did not affect membrane currents, membrane potentials or CBF in the absence of K_ATP_ channel openers.	
			Adenosine (5–50 μM).		Membrane potential measurements from capillary ECs and pericytes on pressurized retina preparations (ophthalmic artery).	Adenosine failed to increase CBF in both ECs and pericytes specific Kir6.1 dominant-negative mice.	
			PNU-377883 (NR).		CBF measurement using laser-Doppler flowmetry through a cranial window over the somatosensory cortex.	Small K_ATP_ current in SMCs isolated from either brain pial arteries or parenchymal arterioles.	
41	Syed et al. (59)	To investigate the role of K_ATP_ channel in regulation of middle meningeal arteries (MMA).	Cromakalim (10 μM).	Rats (*n* = 30)	*Ex vivo*, diameters of intact cerebral arteries and MMA were measured using a video detector in a myograph chamber.	Cromakalim induced a greater vasodilator effect of MMAs compared to cerebral arteries.	K_ATP_ channel activity contributed to the regulation of MMA but not cerebral artery diameter.
			Glibenclamide (1 nM–10 μM).		Smooth muscle membrane potential was measured for both MMA and cerebral arteries.	Glibenclamide and PNU-37883A induced constriction of isolated MMA’s but did not alter cerebral artery diameter.	
			PNU-37883A (10 μM).		*In vivo* two-photon imaging of meningeal blood vessels through a cranial window.	In MMA, glibenclamide caused a membrane potential depolarization in smooth muscle. However, in cerebral artery smooth muscle, membrane potential was not significantly different in the presence or absence of glibenclamide.	
42	Gozalov et al. (60)	To investigate the role of K_ATP_ channels in vasodilation in intracranial arteries by CGRP, NO-donor, glyceryl trinitrate (GTN) and transcranial electrical stimulation.	Glibenclamide (7 mg/kg).	Rats (*n* = NR)	Diameters of dural and pial arteries were measured using a video-analyzer through a cranial window over the parietal skull.	CGRP, GTN and transcranial electrical stimulation induced dilation of dural and pial arteries, *in vivo* and *in vitro*.	Glibenclamide *in vivo* but not *in vitro* inhibited CGRP-induced vasodilation.
						*In vivo*, glibenclamide attenuated CGRP-induced dural artery dilation and transcranial electrical stimulation-induced pial and dural artery dilation.	
			CGRP (0.3 μg/kg).		rCBF were measured over the parietal bone and pial arteries by laser-Doppler flowmetry.	Glibenclamide had no effect on pial or dural vasodilation induced by GTN.	K_ATP_ channels could be involved in the migraine generating effect of CGRP.
			GTN (20 μg/kg).			*In vitro*, glibenclamide did not significantly inhibit the vasodilation induced by GTN and CGRP, respectively.	
43	Taguchi et al. (61)	To investigate whether activation of K_ATP_ channels mediates dilation of cerebral arterioles during hypoxia.	Aprikalim (10^−7^–10^−6^ M).	Rabbits (*n* = 31).	Diameters of cerebral arterioles were measured using a video micrometer through a cranial window over the parietal cortex.	Aprikalim induced dilation of cerebral arterioles which was inhibited by glibenclamide	Dilation of cerebral arterioles in response to hypoxia were mediated by activation of K_ATP_ channels.
			Glibenclamide (10^−6^ M).			Glibenclamide alone had no effect on baseline diameters.	
44	Hariharan et al. (62)	To investigate whether brain capillary pericytes control local blood flow via K_ATP_ channel.	Pinacidil (10 μM).	Mice (*n* = NR)	Diameters of pial arteries were measured through a cranial window.	Barium applied to the cortical surface prior to pinacidil ejection on a pericyte, blocked Kir2.1 channel and abolished the increase in dilation of arterioles and capillary blood flow.	Brain capillary pericytes controlled blood flow through K_ATP_ channel activity.
			Barium (100 μM).		Membrane potential measurements on capillary pericytes.		
45	Simard et al. (63)	To investigate whether SUR1 is an important element in the inflammatory response to subarachnoid hemorrhage (SAH).	Glibenclamide (10 μg/kg and 0.5 μL/h)	Rats (*n* = 35).	The model of SAH involved endovascular puncture of the ICA using a 4-0 filament, produced mild-to-moderate SAH, associated with low mortality.	Critical responses to SAH-inflammation and an increase in barrier permeability, were significantly attenuated by block of SUR1 by glibenclamide, a selective SUR1 inhibitor.	SUR1 was important in the pathophysiology of SAH.
					CBF were measured using Laser Doppler flowmeter affixed to the skull.		
					Shortly after inducing SAH (<15 min), glibenclamide was administrated (loading dose of 10 μg/kg intraperitoneally and then 0.5 μL/h infusion subcutaneously).		
					*In situ* hybridization was used to detect mRNA for *Abcc8* which encodes SUR1.	Immunohistochemistry for SUR1 showed minimal labeling in uninjured controls compared to 24 h after SAH in the inferomedial cortex.	

AD, Alzheimer’s disease, CBF, cerebral blood flow; rCBF, regional cerebral blood flow; CGRP, calcitonin gene-related peptide; EC; endothelial cell; ET-1, endothelin-1; FPI, fluid percussion brain injury; HSP, heat shock protein; KATP, ATP-sensitive potassium; KCI; KATP channel inhibitor KCO; KATP channel opener; MCA, middle cerebral artery; MMA, middle meningeal artery; NO, nitric oxide; NR, not reported; RVL, rostral ventrolateral medulla; SAH, subarachnoid hemorrhage; SMC, smooth muscle cell.

**Table 3:** Summary of clinical studies.

N	Author	Purpose of the study	Substance(s) and dose(s)	Study design	Study population (n)	Method	Main outcome(s)	Conclusion
1	Al-Karagholi et al. (6)	To investigate the effects of levcromakalim and glibenclamide on global CBF (gCBF) and on circumference of extracranial and intracranial arteries.	Levcromakalim (1 mg)	Double-blind, placebo-controlled, three-way crossover design.	Healthy participants (*n* = 15)	Randomization of the participants into 3 different study days, separated by at least 1 week.	Levcromakalim increased global gCBF with 14% and dilated the cerebral arteries.	K_ATP_ channels played an important role in cerebral hemodynamics.
						*Day 1*: Oral glibenclamide followed by levcromakalim infusion.		
						*Day 2:* Oral glibenclamide followed by placebo (isotonic saline) infusion.		
						*Day 3:* Oral placebo (multivitamin pill) followed by placebo (isotonic saline) infusion.		
						The participants underwent 5 MRI sessions: (time points: −20, 60, 120, 160 and 200 min). Administration of oral glibenclamide/placebo infusion at 0 min and administration of levcromakalim/placebo infusion over 20 min at 140 min of the timeline of the study.		
						At each MRI-session, MR angiography and phase-contrast mapping were performed.		
						MR angiography to measure vessels: MCA, MMA and STA		
			Glibenclamide (10 mg).			Phase-contrast mapping to measure gCBF.	Glibenclamide did not alter the cerebral hemodynamics.	
2	Kokoti et al. (64)	To investigate whether glibenclamide attenuates pituitary adenylate cyclase-activating polypeptide-38 (PACAP-38)-induced headache and vascular changes.	PACAP-38 (10 pmol/kg/min).	Double-blind, randomized, placebo-controlled and. Crossover design.	Healthy participants (*n* = 20)	Randomization of the participants into 2 different study days, separated by at least 1 week.	PACAP-38 decreased V_meanMCA_.	PACAP-38 induced vascular changes might be mediated by the SUR2B K_ATP_ channel.
						Intravenous infusion of PACAP-38 over 20 min, immediately followed by either oral glibenclamide or placebo.	Posttreatment with glibenclamide failed to attenuate vascular changes.	
			Glibenclamide (10 mg).			Mean velocity of blood flow in MCA (V_meanMCA_) were measured using transcranial Doppler.		
3	Bayerle-Eder et al. (65)	To investigate whether glibenclamide alters the cerebral and ocular vasodilator response to hypercapnia.	Glibenclamide (5 mg).	Controlled, randomized, double-blind, two-way crossover study	Healthy participants (*n* = 10)	Participants received either oral glibenclamide and intravenous placebo or oral placebo and intravenous insulin.	Hypercapnia caused a significant increase in fundus pulsation amplitude and V_meanMCA_. However, glibenclamide had no effect on hypercapnia-induced hemodynamic responses.	Hypercapnia-induced vasodilation in cerebral and ocular vessels were not mediated by activation of K_ATP_ channels.
						Pulsatile choroidal blood flow was assessed through laser interferometric measurements of fundus pulsation on the participant’s eye.		
						V_meanMCA_ and the ophthalmic artery were measured using Doppler sonography.		
4	Rocha et al. (66)	To investigate whether K_ATP_ channels blockade affects the increase in CBF during hypoxia.	Glibenclamide (5 mg).	NR	Healthy participants (*n* = 9)	After induction of hypoxia, oral glibenclamide was administered.	Hypoxia induced increase in the anterior circulation and were attenuated under K_ATP_ channel blockage.	Activation of K_ATP_ channels modulated vascular tone in the anterior circulation of the brain.
						Blood flow of internal carotid artery and vertebral artery were conducted via Doppler Ultrasound.		
5	Al-Karagholi et al. (3)	To investigate whether opening of K_ATP_ channels causes migraine attack.	Levcromakalim (0.05 mg/min).	Randomized, double-blind, placebo-controlled, crossover study	Migraine patients without aura (*n* = 16)	Randomization of the participants into 2 different study days, separated by at least 1 week.	Levcromakalim increased diameter of STA but had no significant effect on radial artery diameter or V_meanMCA_.	K_ATP_ channels had no significant on V_meanMCA_.
						Intravenous infusion of either levcromakalim or placebo (isotonic saline).		
						V_meanMCA_ were measured using a transcranial Doppler.		
6	Coskun et al. (67)	To investigate the effect of glibenclamide on CGRP-induced headache and vascular changes.	CGRP (1.5 μg/min).	Randomized, double-blind, placebo-controlled, crossover study	Healthy participants (*n* = 20)	Randomization of participants into 2 different study days, separated by at least 1 week.	Glibenclamide had no effect on CGRP-induced headache and vascular changes (decrease in V_MCA_, increase in facial skin blood flow and dilation of STA and radial artery, respectively).	CGRP-induced responses could be mediated by SUR2B K_ATP_ channel.
						Intravenous infusion of CGRP 2 h after oral pretreatment with either placebo (calcium supplement tablet) or glibenclamide.		
						Facial flushing was measured by speckle contrast imager.		
			Glibenclamide (10 mg).			MCA blood flow velocity (V_MCA_) were measured using a transcranial Doppler.		
						Diameters of STA and radial artery were measured using an ultrasonography (Dermascan).		

CBF, cerebral blood flow; gCBF, global cerebral blood flow; CGRP, calcitonin gene-related peptide, KATP, ATP-sensitive potassium; MRI, magnetic resonance imaging; MCA, middle cerebral artery; MMA, middle meningeal artery; NR, not reported; PACAP, pituitary adenylate cyclase activating polypeptide; STA, superficial temporal artery; V_meanMCA_, mean velocity of blood flow in MCA.

### Summary of preclinical studies

K_ATP_ channels are expressed in SMCs (50, 54), ECs (11, 52–54), and pericytes (11, 43, 51, 58, 62). *In-vivo* studies showed that K_ATP_ channel openers (KCOs) dilated pial arteries and pial arterioles measured using a video microscaler through a cranial window in cats (4), rats (35), and pigs/piglets (5, 22–25, 28, 31). The basilar artery was also dilated upon administration of KCOs in rats (44, 45). CBF measured by laser-Doppler flowmeter through a cranial window over the region supplied by the middle cerebral artery (MCA) was increased upon administration of KCOs in mice (46–48). Using patch-clamp electrophysiology, *ex-vivo* studies showed that application of KCOs led to hyperpolarization of pericytes in mice (11) and rats (58), which was inhibited by K_ATP_ channel inhibitor (KCI), glibenclamide. In rats, endothelium removal from cerebral arterioles resulted in decreased dilation in response to administration of KCOs (52) and reduced the vasoconstrictive effect of glibenclamide (53). The majority of preclinical studies showed that glibenclamide reduced the increase in CBF upon KCO administration without altering the baseline CBF nor the baseline vascular tone (11, 28, 29, 31, 34, 35, 40, 53, 54).

### Summary of clinical studies

KCOs have been used in clinical trials for the treatment of angina pectoris, asthma and hypertension. The most common adverse event mentioned during treatment with KCOs was headache (3, 68, 69).

Clinical studies assessed the effect of K_ATP_ channels in cerebral hemodynamic in healthy participants and individuals with migraine using magnetic resonance (MR) angiography and transcranial Doppler. Intravenous infusion of KCO, levcromakalim increased CBF and dilated the MCA, the middle meningeal artery (MMA) and the superficial temporal artery (STA) (3, 6, 70). Glibenclamide did not affect the baseline diameter of intra- and extracerebral arteries (6). In contrast to preclinical studies, glibenclamide failed to attenuate the vasodilation induced by levcromakalim (6) or by other potent endogenous vasodilators including the calcitonin gene-related peptide (CGRP) (67, 71) and the pituitary adenylate cyclase-activating polypeptide (PACAP-38) (64).

## Discussion

The aim of the present study is to systematically review the involvement of K_ATP_ channels in the cerebral vasculature and the contribution of these channels in cerebrovascular hemodynamics. The main findings are that K_ATP_ channels are expressed in cerebral vascular SMCs, ECs and pericytes and play a key role in the regulation of CBF across species (7–12, 72).

The K_ATP_ channel is a hetero-octameric complex consisting of four regulatory sulfonylurea receptor (SUR1, SUR2A or SUR2B) subunits and four pore-forming K^+^ inwardly rectifying (Kir6.1 or Kir6.2) subunits (73). Different compositions of K_ATP_ channel subunits lead to unique functions in distinct tissues (74, 75) (Table 4). K_ATP_ channels, depending on their different subunit composition, are expressed in vascular SMCs and neurons. Of note, in this systematic review, a frequently used KCO, levcromakalim, has a high affinity to the Kir6.1/SUR2B subunit in the vessels (76), while glibenclamide, a non-specific KCI, has a higher affinity to the Kir6.2/SUR1 subunit which is not present in vessels (77).

**Table 4:** Distribution of K_ATP_ channels.

K_ATP_ channels subtypes	Tissue expression
Kir6.1/SUR2B	Smooth muscle
Kir6.2/SUR1	Brain and pancreas
Kir6.2/SUR2A	Cardiac and skeletal muscle
Kir6.2/SUR2B	Smooth muscle

K_ATP_, ATP-sensitive potassium; Kir, K+ inwardly rectifying; SUR, sulfonylurea receptor.

### Expression of K_ATP_ channels

K_ATP_ channels are expressed in SMCs, ECs and pericytes. The latter are contractile cells found on the abluminal surface of the endothelial wall of capillaries (78). Two *ex-vivo* studies using patch-clamp electrophysiology to measure whole cell currents in brain pericytes showed that activation of K_ATP_ channels led to hyperpolarization of pericytes, and this effect was inhibited by glibenclamide (11, 58). K_ATP_ channels expressed in the endothelium of cerebral arteries might be a key component in the regulation of CBF. Endothelium removal of cerebral arterioles significantly affected the response to K_ATP_ channel modulators (52, 53). Endothelium produces numerous vasoactive mediators, including nitric oxide (NO) that influences CBF (10). Impaired endothelial function associated with hypertension (40), diabetes mellitus (35, 52), and aging (45, 46) reduced the impact of KCOs/KCIs. These findings indicate that K_ATP_ channel-induced vasodilation is endothelium-dependent. However, Janigro et al. (54) demonstrated that KCOs caused a pronounced vascular SMC-mediated and a lesser endothelium-dependent vasodilation in rats.

### K_ATP_ channels and cerebral hemodynamics

Administration of synthetic KCOs (Table 1) increased the CBF measured through cranial window using a laser-Doppler flowmeter (11, 40, 44, 46, 48). Whereas, glibenclamide and other synthetic KCIs inhibited the effect induced by KCOs (40, 46, 48). The majority of the preclinical studies showed that glibenclamide did not affect the baseline CBF and the vascular tone measured by laser-Doppler flowmeter (11, 40) except one study which reported that glibenclamide injected in the cisterna magna lowered baseline CBF (38). CBF is dependent on cerebral perfusion pressure (CPP) and cerebrovascular resistance (CVR). The diameter of small arteries and pial arterioles contributes to CVR. In particular, dilation of pial arterioles might increase CBF while constriction of these vessels could decrease CBF (1).

KCOs dilated pial arteries (5, 22–25, 79), pial arterioles (4, 28, 31, 35, 61), the basilar artery (44, 45), and the MCA (50, 52). Here, glibenclamide and other synthetic KCIs reversed this dilation (4, 28, 31, 35, 43–45, 61). Glibenclamide did not affect the baseline diameter of these vessels *in vivo* (28, 29, 31, 34, 35) or *ex vivo* (53, 54). However, in one study, glibenclamide induced constriction of isolated MMAs in the absence of other vasoactive stimuli but did not alter the diameter of cerebral arteries (59).

Inhalation of anesthetics such as isoflurane/sevoflurane or hypoxia caused dilation of cerebral pial arterioles which was inhibited by glibenclamide (32). Adenosine induced dilation of cerebral arterioles in pigs (29) and hyperpolarized retinal pericytes in mice and rats (11, 58) and capillary ECs in mice (11), and administration of glibenclamide inhibited the effects of adenosine. CGRP *in vivo* and *in vitro* induced dilation of dural and pial arteries. Glibenclamide attenuated the effect of CGRP *in vivo*, but not *in vitro* (60). In healthy participants, glibenclamide had no effect on CGRP-induced headache (67).

Clinical studies demonstrated that levcromakalim dilated the MMA, the MCA and the STA in healthy humans (6) and individuals with migraine (3). In contrast to the preclinical studies, glibenclamide failed to attenuate the vascular changes induced by levcromakalim (6), PACAP-38 (64), CGRP (67) or hypercapnia (65). Of note, adenosine, CGRP and PACAP-38 are potent endogenous vasodilators which activate K_ATP_ channels indirectly through adenylate cyclase and protein kinase A phosphorylation (80–82). One study, however, reported that hypoxia increased the anterior circulation of the brain and this effect was attenuated by K_ATP_ channel blockage with glibenclamide (66). The lack of effect of glibenclamide in clinical studies could be attributed to differences in administration routes, metabolic rate and/or tissue expression of K_ATP_ channels across species. Basic mathematical modeling of pharmacokinetics and receptor potencies showed that the dose of glibenclamide used in clinical studies had receptor occupancy of 26% at the migraine relevant K_ATP_ channel subtype Kir6.1/SUR2B (83).

## Limitations and future perspective

The major limitations for the preclinical studies are differences in methodological approaches including subjects, designs, concentrations and formulations of different types of KCOs and KCIs, potentially affecting the reported results (Table 2). Shortcomings of clinical trials assessing the hemodynamics role of K_ATP_ channel are (1) the use of low dose of glibenclamide, (2) including individuals from all age groups, and (3) not evaluating the long-term effect of KCOs or KCIs on cerebral hemodynamics and how endothelial dysfunction interferes with this effect. An additional question is whether K_ATP_ channels are involved in cerebral angiogenesis.

The K_ATP_ channel emerges to be a potential target for numerous pathological conditions such as migraine and ischemic stroke. Recent studies showed that K_ATP_ channel activation caused headache and migraine (3), indicating that KCIs might be a novel therapeutic approach for the treatment of headache and migraine. The fact that targeting K_ATP_ channels did not affect the baseline hemodynamic state, at least based on preclinical studies, is applicable to avoid serious adverse events. Activation of K_ATP_ channels increased CBF after cerebral ischemia in mice (51). More experiments are needed to reveal if KCOs have a clinically meaningful effect on cerebral hypoperfusion during ischemic stroke.

Other findings with direct clinical significance are that glibenclamide attenuated peripheral arterial dilation but failed to affect cerebral hemodynamics indicating an unique biochemical difference between K_ATP_ expressed in cerebral circulation and those expressed in peripheral arteries.

Several scenarios might underlie this difference, including expression of different SUR and Kir6 isoforms, different expression levels, post-translational modifications that render cerebral vascular K_ATP_ channels less sensitive to KCIs and/or existence of other cerebral regulatory mechanisms with higher impact. Western blotting and quantitative PCR could be used to compare the isoforms, expression within cerebral and peripheral arteries. Patch-clamp electrophysiology on isolated SMCs or ECs from the cerebral and peripheral arteries can assess the functional properties and thereby drug sensitivity.

These studies might allow a possible treatment avenue for individuals with hypertension without altering cerebral hemodynamics. Several clinical studies applied KCO to treat hypertension (68, 84–86). However, a common adverse event was headache, most likely due to changes in cephalic hemodynamics. Yet, more selective agonists are needed to avoid adverse events. The next step is the development of a selective KCO to avoid headache when treating hypertension. An agonist with high affinity to the Kir6.1 isoform of K_ATP_ channels could be an applicable candidate.

## Conclusion

Preclinical and clinical data from this systematic review demonstrated that K_ATP_ channels are implicated in the regulation of cerebral hemodynamic. The main findings are that K_ATP_ channels are expressed in cerebral vascular SMCs, ECs and pericytes. KCO increased CBF and dilated cerebral arteries in both preclinical and clinical data. Glibenclamide did not change baseline CBF and cerebral diameter in preclinical studies and did not attenuate the vasodilation induced by KCOs in clinical studies.

## Data availability statement

The original contributions presented in the study are included in the article/supplementary material, further inquiries can be directed to the corresponding author.

## Author contributions

HASD: Writing – original draft, Writing – review & editing. LK: Writing – review & editing, Writing – original draft. MMK: Writing – original draft, Writing – review & editing.

## Glossary

AD: Alzheimer’s disease
CBF: Cerebral blood flow
gCBF: Global cerebral blood flow
rCBF: Regional cerebral blood flow
CGRP: Calcitonin gene-related peptide
ECs: Endothelial cells
ET-1: Endothelin-1
FPI: Fluid percussion brain injury
GTN: Glyceryl trinitrate
HSP: Heat shock protein
K_ATP_: ATP-sensitive potassium
KCI: K_ATP_ channel inhibitor
KCO: K_ATP_ channel opener
Kir: K^+^ inwardly rectifying
L-NAME: N^G^-nitro-L-arginine methyl ester
L-NMMA: N^G^-monomethyl-L-arginine
L-NNA: N^G^-nitro-L-arginine
MCA: Middle cerebral artery
MCAO: Middle cerebral artery occlusion
MMA: Middle meningeal artery
NO: Nitric oxide
PACAP: Pituitary adenylate cyclase-activating polypeptide
RVL: Rostral ventrolateral medulla
SMC: Smooth muscle cell
SNAP: S-nitroso-N-acetylpenicillamine
STA: Superficial temporal artery
SUR: Sulfonylurea receptor
V_MCA_: Velocity of blood flow in MCA
V_meanMCA_: Mean velocity of blood flow in MCA
